# Comparative transcriptome and metabolome analysis of sweet potato (*Ipomoea batatas* (L.) Lam.) tuber development

**DOI:** 10.3389/fpls.2024.1511602

**Published:** 2025-01-07

**Authors:** Yanhui Lin, Yapeng Li, Honglin Zhu, Liqiong Tang, Jing Xu

**Affiliations:** ^1^ Institute of Food Crops, Hainan Academy of Agricultural Sciences/Hainan Key Laboratory of Crop Genetics and Breeding, Haikou, China; ^2^ Sanya Research Institute, Hainan Academy of Agricultural Sciences, Sanya, China

**Keywords:** sweet potato, RNA-seq, metabolome, anthocyanin content, starch content, carotenoid content

## Abstract

**Introduction:**

Sweet potato is an important food, feed and industrial raw material, and its tubers are rich in starch, carotenoids and anthocyanins.

**Methods:**

To elucidate the gene expression regulation and metabolic characteristics during the development of sweet potato tubers, transcriptomic and metabolomic analyses were performed on the tubers of three different sweet potato varieties at three developmental stages (70, 100, and 130 days (d)).

**Results:**

RNA-seq analysis revealed that 16,303 differentially expressed genes (DEGs) were divided into 12 clusters according to their expression patterns, and the pathways of each cluster were annotated. A total of 9118 DEGs were divided into three categories during the same developmental period. A total of 1566 metabolites were detected, which were mainly divided into 12 categories. DEGs and differentially regulated metabolites (DRMs) were significantly enriched in the starch and sucrose metabolism and flavonoid biosynthesis pathways. The DEGs associated with the flavonoid pathway showed greater expression with the development of tubers, with the highest expression occurring at 130 d; chalcone isomerase (CHI) was a key gene associated with 11 flavonoid compounds. The DEGs associated with the starch pathway presented relatively low expression during the development of tubers, with the highest expression occurring at 70 d; UDP-glucose pyrophosphorylase 2 (UPG2) and glycogen synthase (glgA) were able to regulate the key genes of 8 metabolites related to the starch biosynthesis pathway. The anthocyanin content is directly related to changes in the content of peonidin-3-O-(6”-O-feruloyl)sophoroside-5-O-glucoside, which is regulated by the *IbCHI* gene. The abundance of this starch is directly related to changes in the content of D-glucose 6-phosphate and is regulated by the *IbUGP2* and *IbglgA* genes. A total of 14 candidate genes related to starch, carotenoids and anthocyanins in sweet potato tubers, including the *IbCHI*, *IbUGP2* and *IbglgA* genes, were identified via weighted correlation network analysis (WGCNA).

**Conclusion:**

This research provides fresh insights into the levels of anthocyanins, starch, and carotenoids throughout the growth of sweet potato tubers and sheds light on the potential regulatory pathways and candidate genes involved in this developmental progression.

## Introduction

The sweet potato (*Ipomoea batatas* (L.) Lam.) and serves as a significant source for food, feed, and industrial raw cultivars. It is essential for global food security and energy sustainability (Yang et al., 2023). Sweet potatoes are divided into different types such as high starch, vegetable, medicinal and high anthocyanin (Li Y. et al., 2022). Sweet potatoes are rich in nutrients and contain a variety of vitamins, complex carbohydrates, dietary fiber, protein, and other nutrients (Yang et al., 2023). In addition, they contain antioxidants, anthocyanins, carotenoids and other compounds that can scavenge free radicals (Li et al., 2022). Research shows that these substances have antiviral, antioxidant, anti-inflammatory and vasodilatory properties and can also protect the liver, fight cancer, and fight diabetes (Mohanraj and Sivasankar, 2014). Sweet potatoes can be white, yellow, orange or purple due to varying levels of phenolic compounds and pigments (Xu et al., 2018). Compared with cereal crops, sweet potato has become the main raw material crop for starch production because of its strong photosynthetic ability and high starch yield per unit area (Xu et al., 2018). Starch is one of the main components of sweet potato tubers, accounting for 50 to 80% of the dry matter content (Wang et al., 2020). Because sweet potato is a functional food rich in carbohydrates, trace elements, carotenoids, anthocyanins and other nutrients, the use of genetic engineering technology to improve its yield and quality has become a current research hotspot.

Transcriptome analysis is essential for understanding genome function and identifying differential gene expression and is crucial for investigating plant growth and development processes (Yang and Wei, 2015). Metabolomics complements genomics, RNA-seq, and proteomics, with a focus on studying the downstream metabolites of the genome as a whole. This approach allows for the examination of related metabolic pathways and networks, providing valuable insights into the overall metabolic processes within an organism (Tugizimana et al., 2018). It also intuitively reflects the biochemical pathways and potential molecular mechanisms of organisms. Recent advancements in RNA-seq and metabolomics technologies have offered useful tools and methodologies for revealing molecular characteristics and identifying candidate genes associated with plant growth, development, and fruit quality. By analyzing clusters of metabolites and genes with the same change trend during the fruit development stages of cashew trees (*Anacardium occidentale* L.), 17 genes involved in phosphatidylinositol (PI) synthesis were found, and the transcription factor *WRKY11*, which can promote the synthesis of PI, was also identified (Zhao et al., 2023). RNA-seq and metabolomic technologies have been used to study the accumulation patterns and transcriptional changes of metabolites in the late-maturing mango variety Katemang at different stages of fruit development and to construct a regulatory network related to fruit ripening (Datir and Regan, 2023). By integrating transcriptome and metabolome analyses of various leaf crown grape species, researchers identified 9 genes potentially regulating 9 phenolic acids and three genes involved in the control of 16 flavonoids (Han et al., 2023). The transcriptomes and metabolomes of different tissue samples throughout the rice growth period were used to construct a regulatory network throughout the growth period, and a new network of lignin metabolism was screened through known functional genes (Yang et al., 2022). Multiomics analysis of coconuts revealed that alterations in GA (gibberellic acid) metabolism and natural variations in GA20-oxidase (GA20ox) play crucial roles in determining the disparity in plant height between tall and dwarf coconut species (Wang et al., 2021).

Sweet potatoes can improve dietary intake and are therefore beneficial to human health (Li Y. et al., 2022). The complexity of the sweet potato genome, which is a highly heterozygous hexaploid crop, presents challenges for further research. However, the completion of sweet potato hexaploid genome sequencing has significantly accelerated progress in sweet potato research (Yang et al., 2017; Yan et al., 2022). Most previous studies have focused on the flavonoid metabolome in sweet potato and the multiomics analysis of drought and salt tolerance (Zhang et al., 2022; Zhou et al., 2012; Huang et al., 2022; Zhao et al., 2022). Research on the anthocyanin, starch and carotenoid contents of the tuber is lacking. In this study, three diverse sweet potato varieties were subjected to RNA-seq and metabolomic analyses at three stages of tuber development. Cluster analysis of the differentially expressed genes (DEGs) and differentially regulated metabolites (DRMs), Kyoto Encyclopedia of Genes and Genomes (KEGG) enrichment analysis, and transcription factor expression analysis were carried out. Coexpression analysis helped identify key genes associated with anthocyanin, starch, and carotenoid contents during sweet potato tuber development. These findings provide novel insights into anthocyanin, starch, and carotenoid dynamics in sweet potato tuber formation, revealing potential regulatory pathways and candidate genes influencing this developmental process.

## Materials and methods

### Plant materials

**Figure 1:**
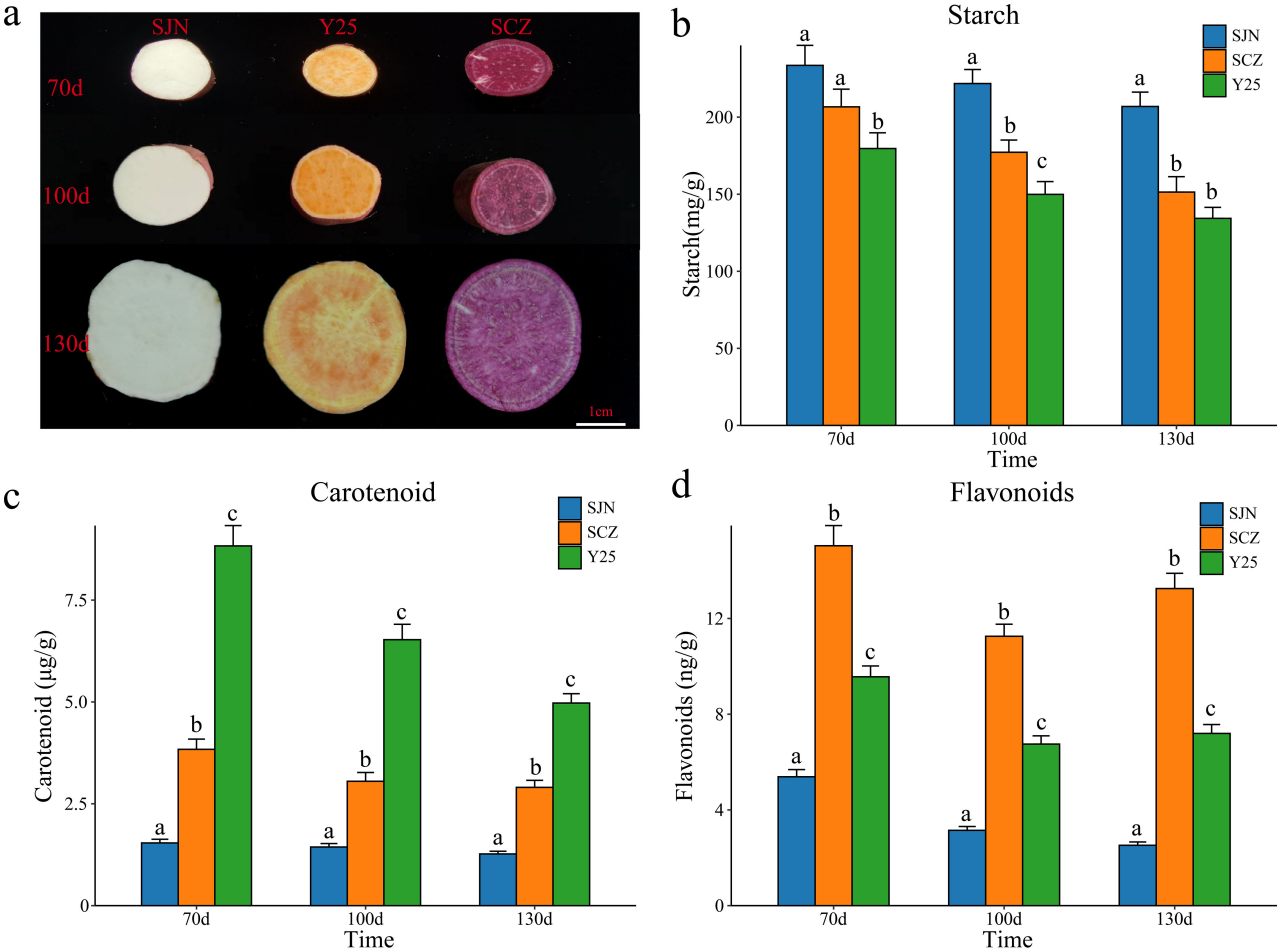
**(A)** Tuber phenotypes of sweet potatoes SJN, SCZ and Y25 at 70, 100 and 130 d; bar = 1 cm. **(B)** Amylum contents of SJN, SCZ and Y25 at 70 100 and 130d. The results are displayed as the mean ± standard deviation (n=3, different lowercase letters indicate that different varieties or stages periods reach significant levels, P < 0.05). **(C)** Carotenoid contents of SJN, SCZ and Y25 at 70, 100 and 130d. The results are displayed as the mean ± standard deviation (n=3, different lowercase letters indicate that different varieties or stages periods reach significant levels, P < 0.05). **(D)** Flavonoid content at 70, 100 and 130 d in SJN, SCZ and Y25. The results are displayed as the mean ± standard deviation (n=3, different lowercase letters indicate that different varieties or stages periods reach significant levels, P < 0.05).

SJN (Sanjiaoning, a local, white-fleshed sweet potato variety from Hainan), SCZ (Shanchuanzi, a purple-fleshed sweet potato introduced from Japan) and Y25 (Yanshu 25, an orange-fleshed sweet potato bred by the Yantai Academy of Agricultural Sciences) were selected for planting in Hainan Province, Chengmai County, Yongfa Town. The area of the plot was 7 m^2^ (3.5 m×2 m), and each plot had 3 ridges, which were measured 3 times. The ridge width was 90 cm, the height was 40 cm, the ridge spacing was 40 cm, and the plant spacing was 20 cm. The slips were planted on October 11, 2022, tubers were harvested on March 4, 2023, and the plants were managed in the same manner as conventional field cultivation. Sampling occurred at 70, 100, and 130 d after planting (**Figure 1A**). Twelve replicates were obtained for each sample: 3 for RNA-seq sequencing, 3 for metabolome sequencing, 3 for physiological indicator analysis, and 3 for qRT−PCR.

### Determination of anthocyanin, starch and carotenoid contents

The sweet potato tuber samples were removed and stored at -80°C. The physiological indicators were determined according to the kit method of Suzhou Mengxi Biomedical Technology Co., Ltd., and the enzyme labeling method was used to determine the carotenoid and starch contents (Zavřel et al., 2018). The anthocyanin content was determined via a Waters UPLC I-Class Plus (Waters, USA) tandem Q Exactive high-resolution mass spectrometer (Thermo Fisher Scientific, USA) (Sigan et al., 2024). Three biological replicates were performed for each indicator.

### RNA-seq and analysis

RNA-seq was conducted by Beijing Biomics Biotech Co., Ltd., in Beijing, China. Following the generation of the initial sequencing data, fastp software (version 0.23.4) was used to eliminate adapter sequences and filter out low-quality sequences (with Q<=20) and those with more than 10% N sequences to yield clean reads suitable for further analysis (Chen et al., 2018). HISAT2 was subsequently utilized to align the clean reads to the sweet potato reference genome available at http://sweetpotato.plantbiology.msu.edu/ (Pertea et al., 2016). To characterize expression levels, fragments per kilobase of transcript per million mapped reads (FPKM) were utilized. The gene read count (raw count) was used for differential expression analysis, and edgeR software was used to compute the P value and fold change. P values ≤ 0.05 and |log_2_fold change|>1 were used to identify DEGs (Liu et al., 2021). DEGs were annotated for gene function via the KEGG database (http://www.genome.jp/kegg/). The protein sequences of the DEGs were analyzed and predicted via PlantTFDB (http://planttfdb.cbi.pku.edu.cn/prediction.php#) to identify differentially expressed transcription factors.

### UPLC−MS analysis

Metabolome sequencing was conducted by Beijing Biomics Biotech Co., Ltd., in Beijing, China. For metabolite separation and detection, a Waters UPLC I-Class Plus coupled with a Q Exactive high-resolution mass spectrometer (Thermo Fisher Scientific, USA) was utilized. The mass spectrum scanning range was set at 125–1500 for positive ions and 100–1500 for negative ions. The parameters included a first-level resolution of 70,000, an AGC of 1e6, and a maximum injection time (IT) of 100 ms. Data processing was performed via Analyst 1.6.3 software. Qualitative and quantitative analysis of metabolites in the samples was carried out by leveraging the mzCloud database, ChemSpider online database, and local metabolism database to interpret and identify the metabolites through mass spectrometry.

### Metabolome analysis

Principal component analysis (PCA) was conducted on each sample via the R language PCAtools package (version 2.19.0) based on the metabolite content data matrix. Metabolites were classified, and pathways were functionally annotated via the KEGG database to identify the main biochemical metabolism pathways and signal transduction pathways involved. Differentially abundant metabolites were defined based on criteria that included a |log_2_fold change|>1 and P values ≤ 0.05. Annotation of gene function for differentially abundant metabolites was performed via the KEGG database (http://www.genome.jp/kegg/).

### Joint analysis of RNA-seq and metabolome data

DEGs and DRMs were simultaneously mapped onto the KEGG pathway diagram to pinpoint enriched pathways within both groups. The correlations between metabolites and genes are depicted through a network diagram. For each pathway, DEGs and differentially abundant metabolites with a Pearson correlation coefficient exceeding 0.95 and a p value lower than 0.01 were selected for visualization. Additionally, the expression patterns of fundamental genes within gene pathways were scrutinized.

### WGCNA

The WGCNA package (version 1.73) in R language was used to conduct coexpression analysis of the gene expression profiles of the DEGs via the dynamic branch cutting method (Langfelder and Horvath, 2008). A weighting coefficient of β=16 was chosen for the analysis. The blockwise module automatic network construction function was employed to generate the network, resulting in multiple modules with varying gene counts. By applying standards such as minModuleSize = 30 and Merge Cut Height = 0.25, modules with 0.75 similarity were merged. The correlation coefficient between the module’s characteristic vector ME (module eigengene) and compound content or treatments at different time points was calculated. Visualization of the coexpression networks was conducted via Cytoscape software (version 3.10.1) (Shannon et al., 2003).

### qRT−PCR

Total RNA extraction was carried out via the E.Z.N.A. The Plant RNA Kit was obtained from Omega Bio-Tek (Doraville, GA, USA). First-strand cDNA synthesis was performed through reverse transcription using 1 μg of isolated RNA with the PrimeScript™ RT Kit containing gDNA Erasure from Takara Bio Inc. in Shiga, Japan. Primers specific to the gene sequence were designed via Primer3 online (https://primer3.ut.ee/) (Thornton et al., 2011). Subsequently, qRT−PCR analysis was conducted via a Line-Gene9600 fluorescence quantitative PCR instrument from Hangzhou Bioer Technology Co., Ltd. in China, with SYBR Green from Takara Bio, Inc. The reaction program for qRT−PCR analysis included an initial denaturation step at 95°C for 30 s, followed by 35 cycles of denaturation at 95°C for 5 s, annealing at 60°C for 5 s, and extension at 72°C for 35 s. The results were quantified via the 2^-ΔΔCt^ method (Maren et al., 2023). The internal reference gene utilized was *Ibactin*, and each gene was subjected to three biological replicates. A list of all the primers used in this study can be found in **Supplementary Table S1**.

## Results

### Determination of anthocyanin, starch and carotenoid contents during SJN, SCZ and Y25 tuber development

The content and quality of anthocyanins, starch and carotenoids are important indicators for identifying the quality of sweet potato and determining its taste and use. The contents of different sweet potato tubers at the same developmental stage varied greatly (**Figure 1**). We measured the anthocyanin, starch and carotenoid contents of SJN, SCZ and Y25 tubers at 70, 100 and 130 d of development (**Figure 1**). At all stages of tuber development for the three varieties, SJN presented the highest starch content, which was significantly greater than that of SCZ and Y25. As the sweet potato tubers developed, the starch content gradually decreased (**Figure 1B**). Y25 presented the highest carotenoid content, which was significantly greater than that of SJN and SCZ. As the sweet potato tubers developed, the carotenoid content gradually increased and reached a maximum value at 130 d (**Figure 1C**). SCZ had the highest anthocyanin content, which was significantly greater than that of SJN and Y25. As sweet potato tubers developed, the anthocyanin content decreased slightly. SJN had the lowest content at 130 d, and SCZ and Y25 had the lowest content at 100 d (**Figure 1D**). To further explore the key genes and metabolites related to anthocyanin, starch and carotenoid contents during sweet potato tuber development, RNA-seq and metabolome sequencing were performed on the tubers of SJN, SCZ and Y25 at 70, 100 and 130 d of development.

### RNA-seq analysis

**Figure 2:**
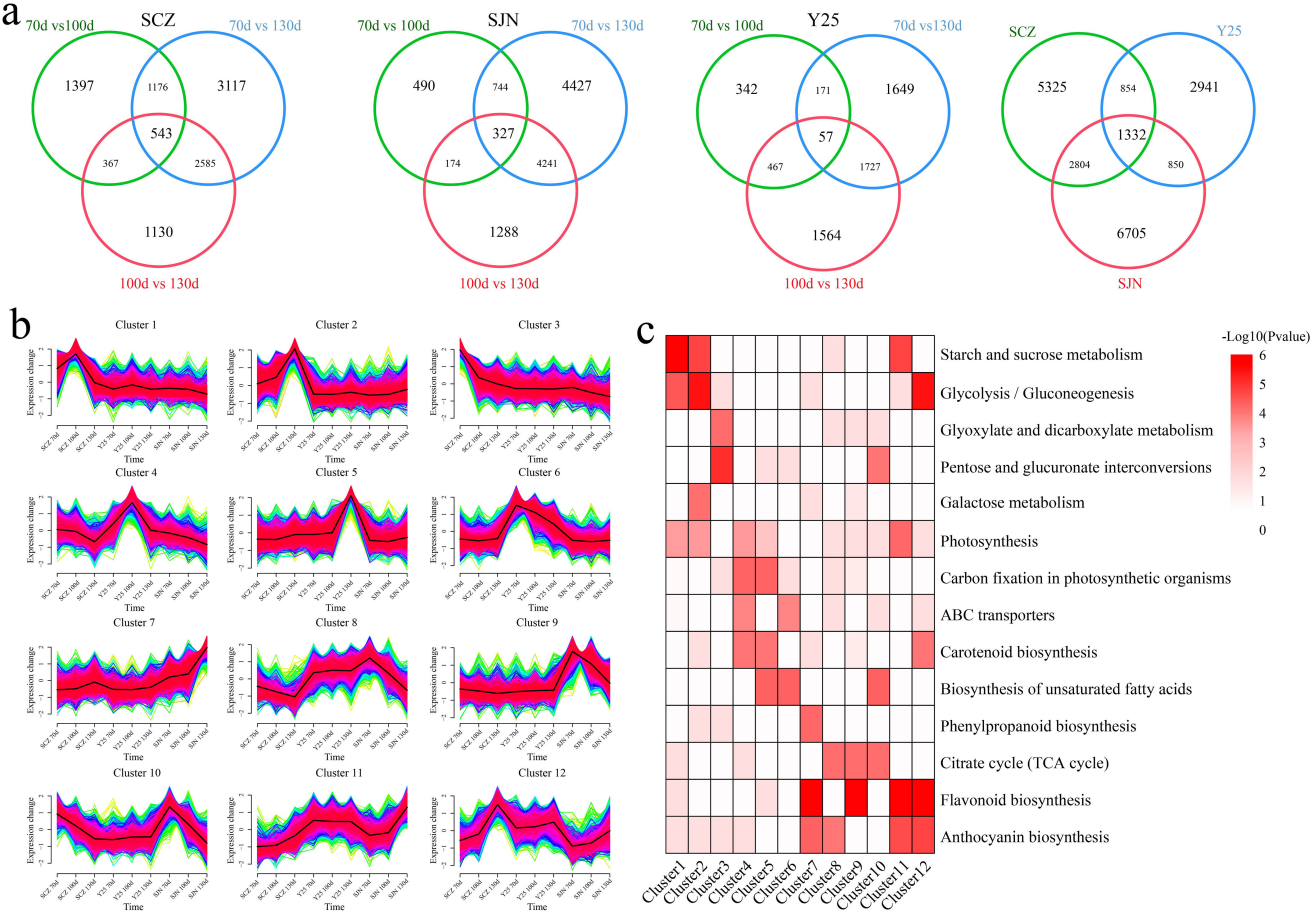
**(A)** Differential expression analysis of the same material at different stages of tuber development. **(B)** Clustered line plots of common and unique DEGs expression patterns within the material. **(C)** Common and unique DEGs clustered within the material KEGG pathway annotation.

A total of 232.38 Gb of clean data were generated from the RNA-seq analysis of 27 samples collected from 3 cultivars at 3 stages. Each sample yielded a minimum of 7.14 Gb of clean data. The Q30 score ranged between 93.60% and 95.06%. A total of 91.02% to 95.37% of the reads were mapped to the reference genome, and the percentage of unique mapped reads ranged from 89.55% to 92.72% (**Supplementary Table S2**). The correlation between replicate samples of the same biological entity was strong, with correlation coefficients ranging from 0.94 to 1.00. PCA and correlation analyses revealed consistent results, with all the replicates closely clustered together, indicating the reliability and reproducibility of the transcriptome data and laying the groundwork for subsequent analyses (**Supplementary Figure S1**). First, differential expression analysis was performed on the same material at different developmental stages, and 10315 DEGs were identified during the three time periods of SCZ development, including 543 common DEGs, 1397 unique DEGs at 70 d vs. 100 d, 3117 unique DEGs at 70 d vs. 130 d, and 1130 unique DEGs at 100 d vs. 130 d (**Figure 2A**). A total of 11,691 DEGs were identified during the three time periods of SJN development, including 327 common DEGs, 490 unique DEGs at 70 d vs. 100 d, 4427 unique DEGs at 70 d vs. 130 d, and 1288 unique DEGs at 100 d vs. 130 d. A total of 5977 DEGs were identified during the three time periods of Y25 development, including 57 common DEGs, 342 unique DEGs at 70 d vs. 100 d, 1649 unique DEGs at 70 d vs. 130 d, and 1564 unique DEGs at 100 d vs. 130 d. A total of 5325 unique DEGs were identified during the three time periods of SCZ development, 2941 unique DEGs were identified during the three time periods of Y25 development, and 6705 unique DEGs were identified during the three time periods of SJN development, with 1332 common DEGs. The 16,303 shared and unique DEGs were divided into 12 clusters according to their expression patterns, and the pathways of each cluster were annotated (**Figures 2B, C**). Clusters 1, 2 and 3 exhibited differential expression only during the three developmental stages of SCZ. Clusters 1 and 2 both exhibited significantly upregulated expression, with notable annotations in the starch and sucrose metabolism, glycolysis/gluconeogenesis, and photosynthesis pathways. Clusters 4, 5 and 6 only presented differential expression in the three developmental stages of Y25 tubers. Clusters 4 and 5 were upregulated, with significant annotations in the photosynthesis, carbon fixation in photosynthetic organisms, and carotenoid biosynthesis pathways. Clusters 8 and 9 were downregulated in all three stages of SJN tuber development and were significantly annotated in the citrate cycle (TCA cycle) pathway. Cluster 10 was downregulated in three stages of tuber development in SCZ and SJN, with significant annotations for pentose and glucuronate interconversions, biosynthesis of unsaturated fatty acids and the citrate cycle (TCA cycle) pathway. Clusters 11 and 12 were upregulated in the three stages of tuber development in SCZ and SJN, with significant annotations for starch and sucrose metabolism, glycolysis/gluconeogenesis, photosynthesis, flavonoid biosynthesis and the anthocyanin biosynthesis pathway.

**Figure 3:**
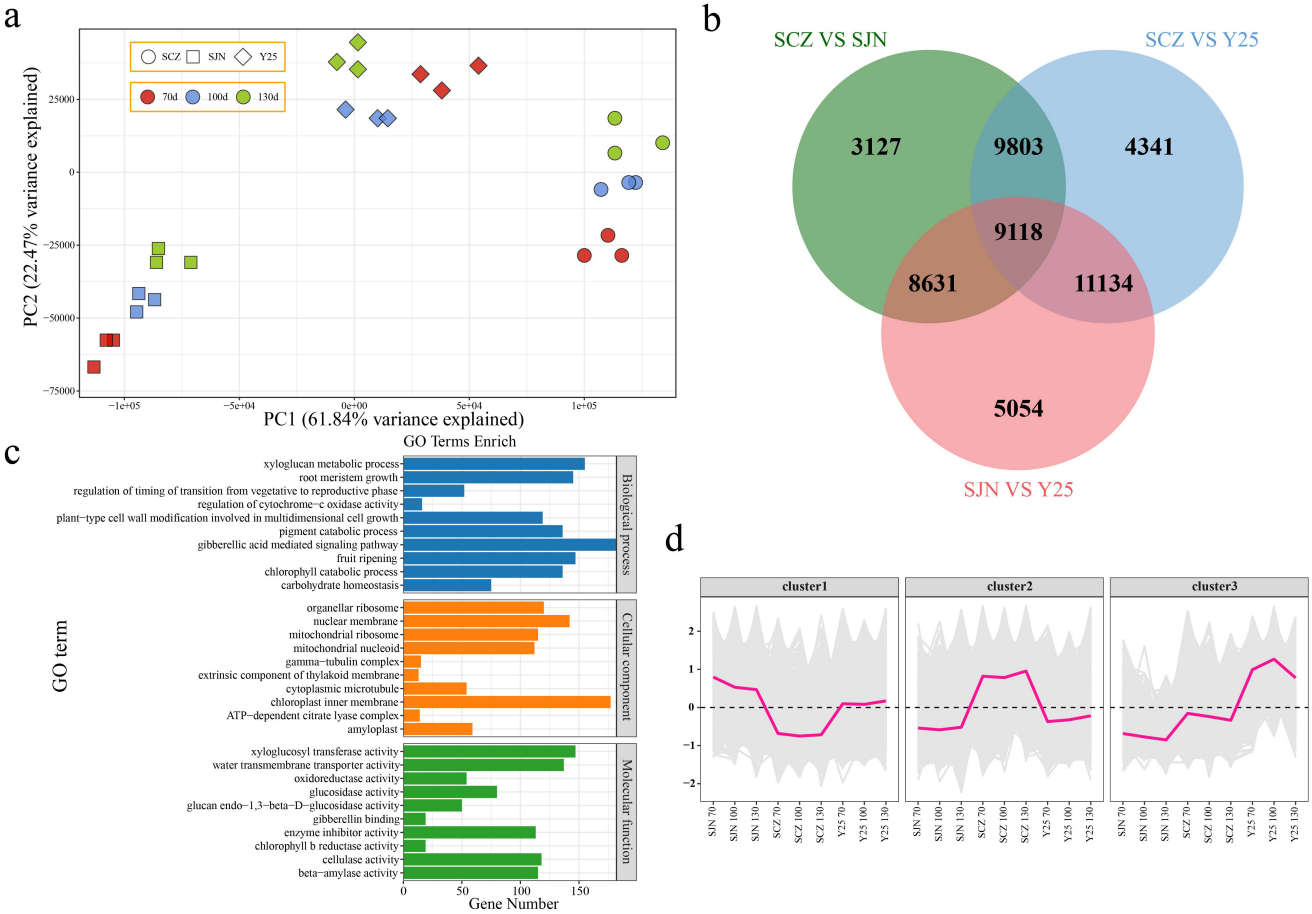
**(A)** PCA of RNA-seq data. **(B)** Venn diagram of DEGs between materials. **(C)** GO enrichment analysis of DEGs among materials. **(D)** Line chart of cluster expression trends of DEGs among materials.

PCA revealed that the variations among the cultivars exceeded the variances observed between different developmental stages within each material. This finding indicates that the discrepancies in gene expression across the different cultivars primarily accounted for the distinct developmental patterns observed in the three cultivars (**Figure 3A**). Therefore, we conducted a differential analysis between the cultivars. There were 30,679 DEGs between SCZ and SJN, 34,396 DEGs between SCZ and Y25, 33,937 DEGs between SJN and Y25, and a total of 9,118 DEGs between the three cultivars (**Figure 3B**). GO functional enrichment analysis was subsequently conducted to validate the functions of the DEGs. The analysis revealed enrichment in various biological processes, including xyloglucan metabolic processes, plant-type cell wall modifications facilitating multidimensional cell growth, fruit ripening, gibberellic acid-mediated signaling pathways, chlorophyll catabolic processes, pigment catabolic processes, regulation of transitions from vegetative to reproductive phases, and carbohydrate homeostasis (**Figure 3C**). The 9118 differentially expressed genes (DEGs) shared across the three cultivars and developmental stages were categorized based on their expression patterns. Cluster 1 presented lower expression in SCZ than in SJN and Y25 at all three developmental stages, with no significant changes during sweet potato tuber development. Cluster 2 displayed higher expression in SCZ than in SJN and Y25, without significant variation across tuber development stages. Cluster 3 presented greater expression in Y25 than in SJN and SCZ. Although the expression levels of SJN and SCZ decreased slightly during sweet potato tuber development, the expression in Y25 initially increased but then decreased (**Figure 3D**).

### Metabolome analysis

**Figure 4:**
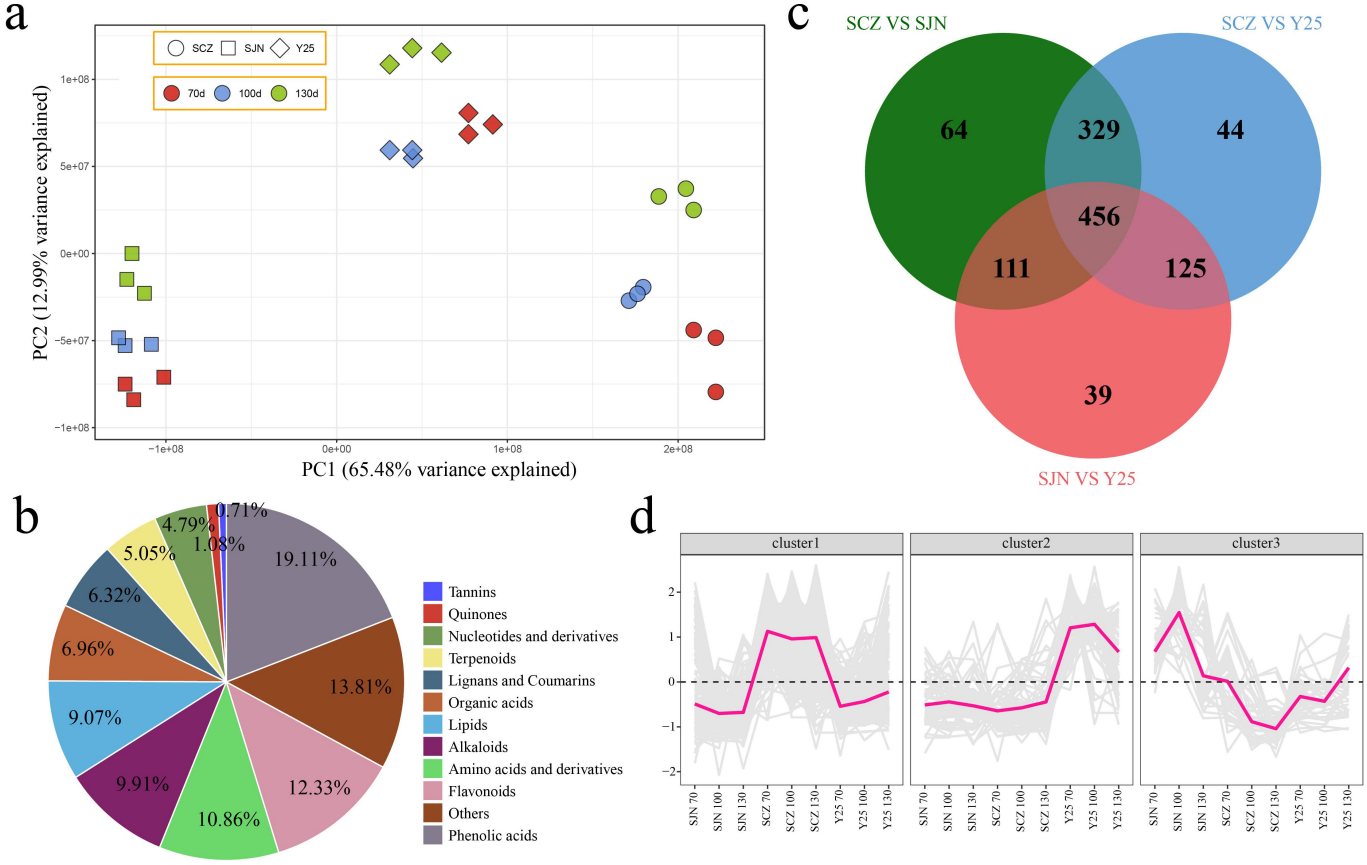
**(A)** PCA of the metabolome data. **(B)** Percentage pie chart of different categories of metabolites. **(C)** Venn diagram of DRMs between materials. **(D)** Line chart of cluster expression trends of DRMs among materials.

UPLC−MS analysis revealed a total of 1566 metabolites across the three developmental stages of the three cultivars. PCA demonstrated that the variations among the cultivars were more pronounced than the variances within the cultivars, which aligns with the results observed in the RNA-seq analysis (**Figure 4A**). To understand the classification and functional attributes of the various metabolites, an annotation process was conducted. The identified metabolites were categorized into 11 groups based on their characteristics. Phenolic acids constituted the largest portion at 19.11%, followed by unclassified metabolites at 13.81%, flavonoids at 12.33%, amino acids and derivatives at 10.86%, alkaloids at 9.91%, lipids at 9.07%, organic acids at 6.96%, lignans and coumarins at 6.32%, terpenoids at 5.05%, nucleotides and derivatives at 4.79%, quinones at 1.08%, and tannins in the lowest proportion, representing only 0.71% of the metabolites (**Figure 4B**). We conducted a difference analysis between cultivars. There were 960 DRMs between SCZ and SJN, 954 DRMs between SCZ and Y25, 731 DRMs between SJN and Y25, and a total of 456 DRMs among the three cultivars (**Figure 4C**). The 456 DRMs shared between cultivars in the three developmental stages can be divided into three categories. Throughout the three developmental stages, the abundance of metabolites in Cluster 1 was greater in SCZ than in SJN and Y25, with consistent levels observed across the development of sweet potato tubers. In Cluster 2, Y25 presented higher levels of these metabolites than did SJN and SCZ, and the content remained relatively stable in SJN and SCZ throughout the developmental stages. The metabolite content in Cluster 3 was highest in SJN, with a pattern of initial increase and subsequent decrease as sweet potato tubers developed. In SCZ, the content was lowest and consistently decreased with increasing tuber development. In Y25, the content initially decreased, followed by an increase (**Figure 4D**).

### Joint analysis of RNA-seq and metabolome data

**Figure 5:**
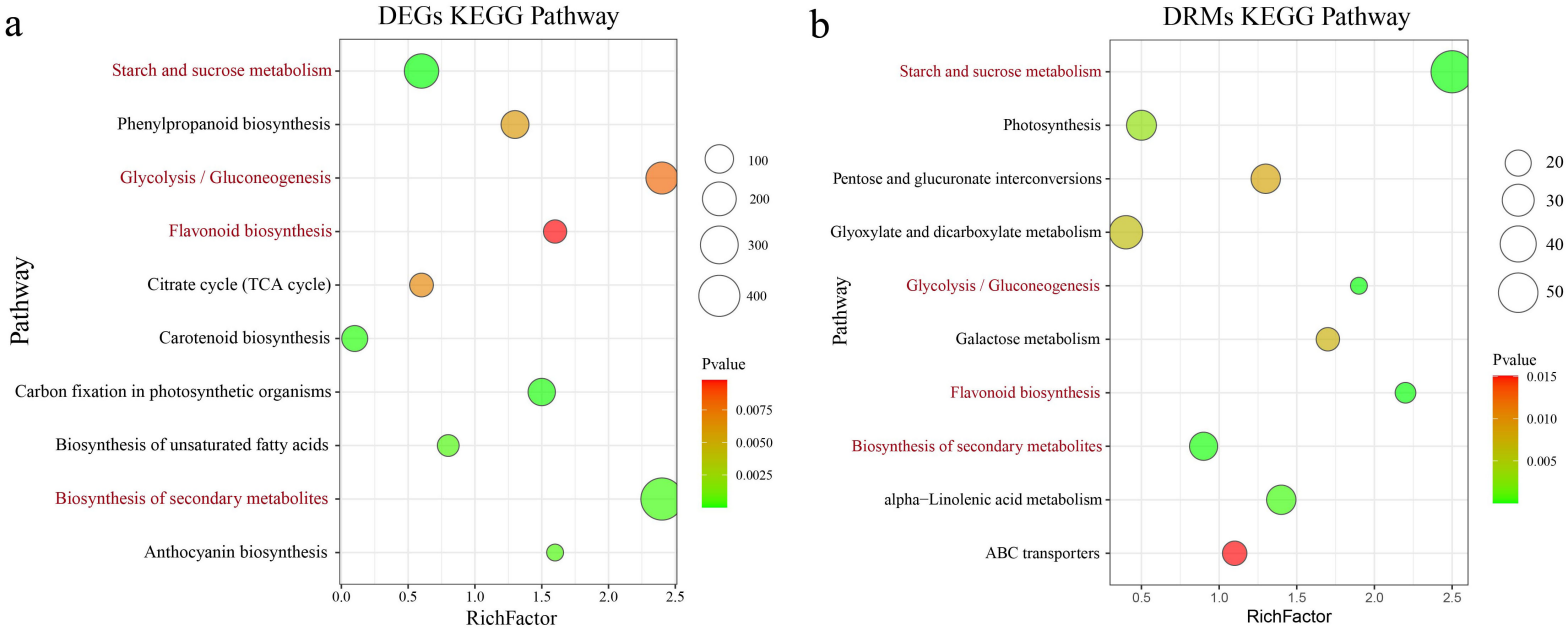
**(A)** Bubble plot illustrating the results of the KEGG enrichment analysis of DEGs across different materials. **(B)** Bubble plot displaying the results of the KEGG enrichment analysis of DEGs among different materials. Red pathways represent coenrichment in both the RNA-seq and metabolome data.

Through integrated analysis of the transcriptome and metabolome, the relationships between DEGs and DRMs in the tubers of various sweet potato cultivars were systematically investigated. The DEGs were significantly enriched in starch and sucrose metabolism, carotenoid biosynthesis, secondary metabolite biosynthesis, anthocyanin biosynthesis, biosynthesis of unsaturated fatty acids, carbon fixation in photosynthetic organisms, phenylpropanoid biosynthesis, glycolysis/gluconeogenesis, flavonoid biosynthesis and the citrate cycle (TCA cycle) (**Figure 5A**). DRMs were significantly enriched in various metabolic pathways, including starch and sucrose metabolism, biosynthesis of secondary metabolites, glycolysis/gluconeogenesis, flavonoid biosynthesis, photosynthesis, alpha-linolenic acid metabolism, galactose metabolism, glyoxylate and dicarboxylate metabolism, pentose and glucuronate interconversions, and ABC transporter pathways (**Figure 5B**). Starch and sucrose metabolism, the biosynthesis of secondary metabolites, glycolysis/gluconeogenesis, and flavonoid biosynthesis were identified as coenriched pathways between DRMs and DEGs. These pathways are crucial and may play significant roles in sweet potato metabolism.

**Figure 6:**
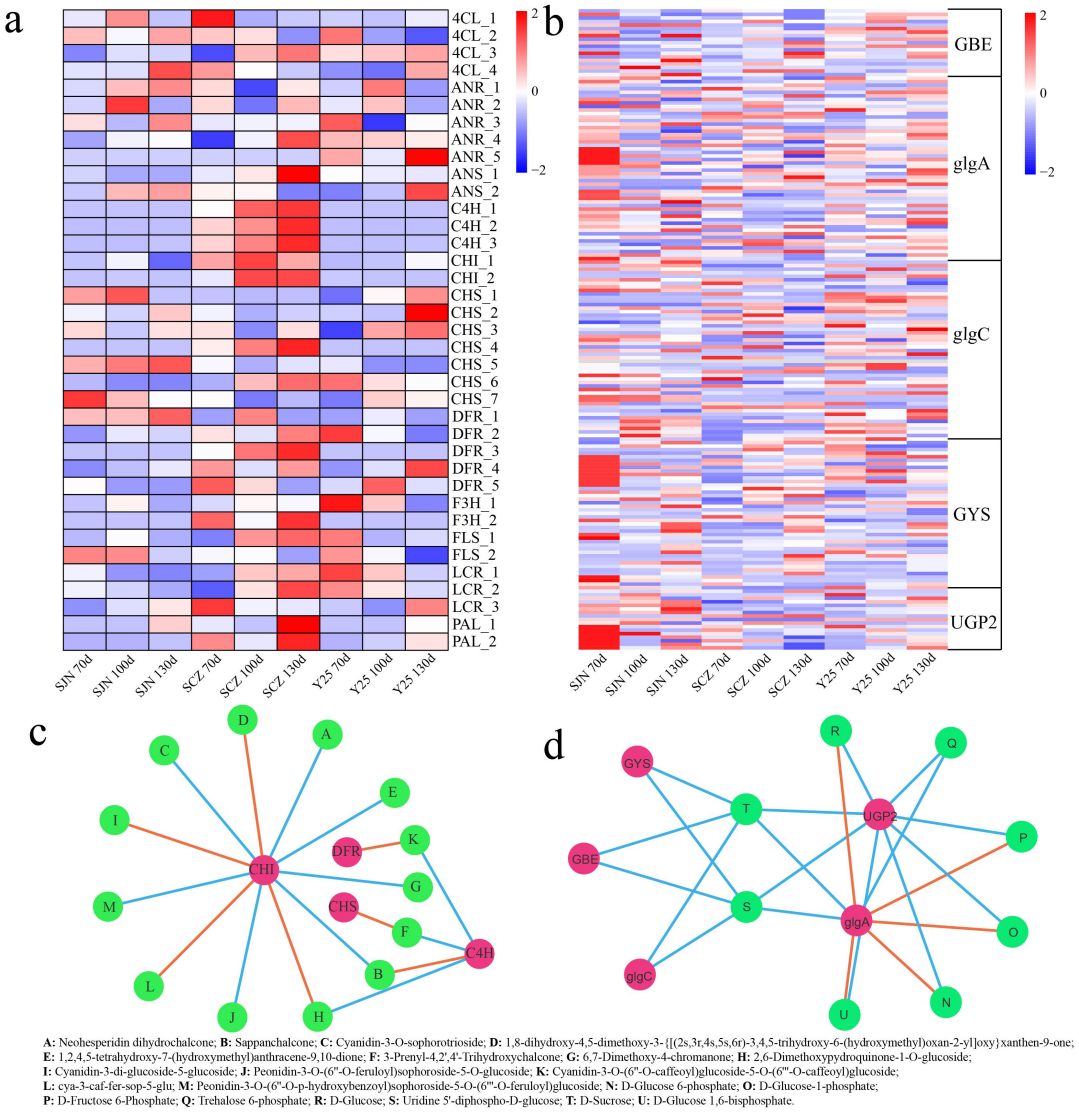
**(A)** Expression heatmap of flavonoid synthesis pathway DEGs. **(B)** Expression heatmap of starch synthesis pathway DEGs. **(C)** Correlation network between flavonoid synthesis pathway genes and flavonoid content. Orange indicates a negative correlation, whereas blue indicates a positive correlation in the constructed network between DEGs and DRMs. **(D)** Correlation network between starch synthesis pathway genes and flavonoid content. Orange indicates a negative correlation, whereas blue indicates a positive correlation in the constructed network between DEGs and DRMs.

Starch and sucrose metabolism as well as flavonoid biosynthesis pathways were found to be closely linked to starch and anthocyanins. DEGs associated with the flavonoid biosynthesis and starch biosynthetic pathways were identified in this study. Compared with those in SJN and Y25, the expression levels of DEGs related to flavonoid biosynthesis, including genes such as cinnamate 4-hydroxylase (C4H), chalcone isomerase (CHI), dihydroflavonol 4-reductase (DFR), leucoanthocyanidin reductase (LAR), and phenylalanine ammonia lyase (PAL), were notably greater in SCZ, with peak expression observed at 130 d (**Figure 6A**). DEGs associated with biosynthesis of starch, such as glycogen synthase (glgA), glycogen synthase (GYS) and UDP-glucose pyrophosphorylase 2 (UPG2), presented increased expression in SJN, with the highest expression occurring at 70 d (**Figure 6B**). To determine the correlation between DEGs and DRMs participating in these pathways, a network linking DEGs and DRMs was developed employing the screening criteria of a Pearson correlation coefficient (PCC) ≥ 0.95 and significance at P < 0.01. Thirteen flavonoid compounds were found to be associated with four genes in the constructed network. CHI was the key gene associated with 11 flavonoid compounds, and 7 were positively correlated (neohesperidin dihydrochalcone, sappanchalcone, cyanidin-3-O-sophorotrioside, 1,2,4,5-tetrahydroxy-7-(hydroxymethyl)anthracene-9,10-dione, 6,7-dimethoxy-4-chromanone, peonidin-3-O-(6’’-O-feruloyl)sophoroside-5-O-glucoside and peonidin-3-O-(6’’-O-p-hydroxybenzoyl) sophoroside-5-O-(6’’’-O-feruloyl)glucoside), 4 were negatively correlated (1,8-dihydroxy-4,5-dimethoxy-3-{[(2 s,3r,4 s,5 s,6r)-3,4,5-trihydroxy-6-(hydroxymethyl)oxan-2-yl]oxy}xanthen-9-one, 2,6- dimethoxypydroquinone-1-O-glucoside, cyanidin-3-di-glucoside-5-glucoside and cya-3-caf-fer-sop-5-glu) (**Figure 6C**). C4H controlled 4 flavonoid compounds, including 2 positively and 2 negatively regulated compounds. PAL and chalcone synthase (CHS) positively regulated 1 flavonoid compound. In the starch biosynthesis pathway, UGP2 positively regulated 8 metabolites (D-glucose 6-phosphate, D-glucose-1-phosphate, D-fructose 6-phosphate, trehalose 6-phosphate, D-glucose, uridine 5’-diphospho-D-glucose, D-sucrose and D-glucose 1,6-bisphosphate). glgA was also able to control 8 starch compounds, including 3 positively and 5 negatively regulated compounds (**Figure 6D**).

**Figure 7:**
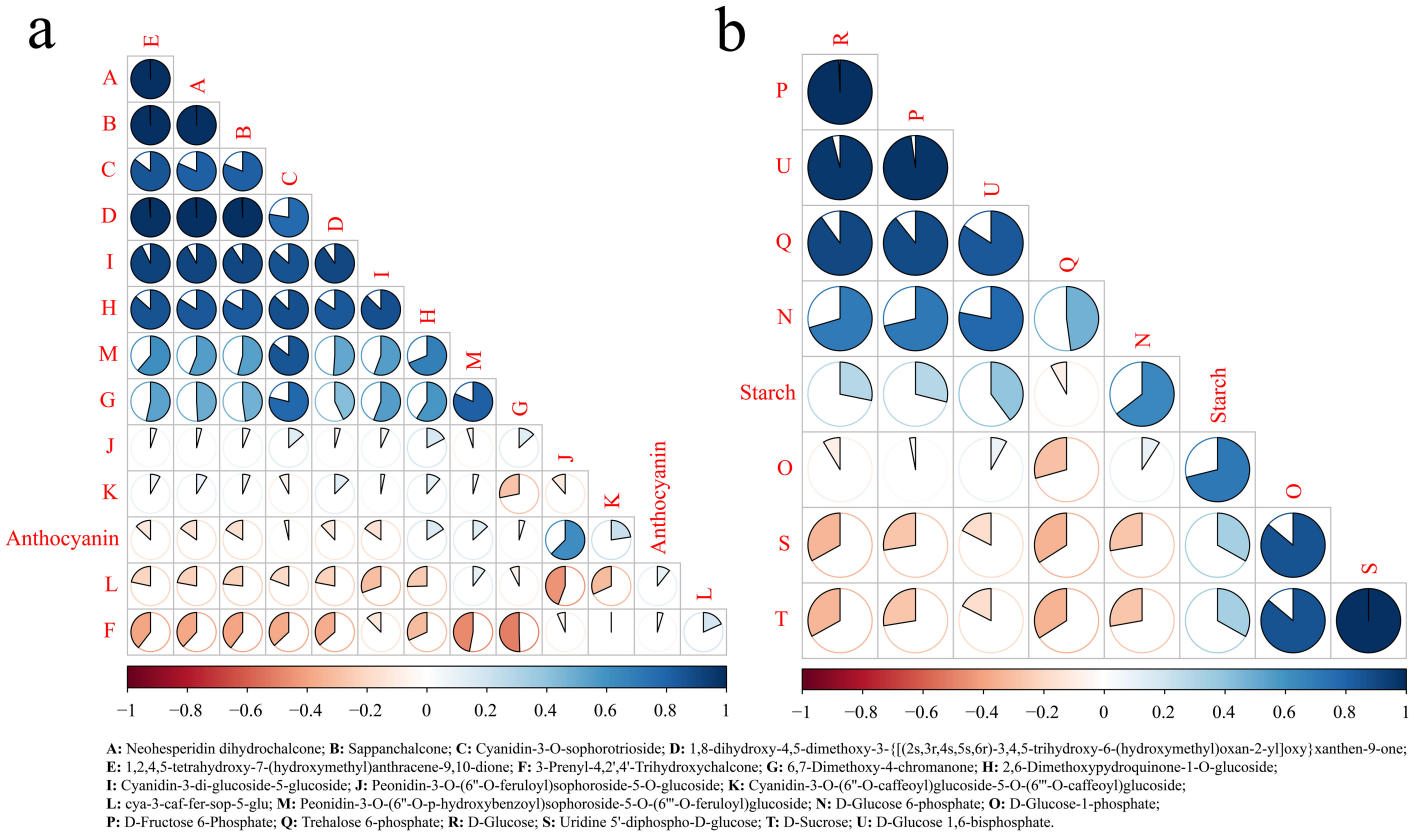
Correlation analysis of flavonoids with metabolites of starch metabolic pathways. **(A)** Correlation analysis between flavonoid synthesis pathway metabolites and anthocyanin. **(B)** Correlation analysis between starch synthesis pathway metabolites and starch.

To further determine the most relevant metabolites of starch and anthocyanins, correlation analysis between different metabolites and starch and anthocyanin contents in the starch and sucrose metabolism and flavonoid biosynthesis pathways was carried out. Neohesperidin dihydrochalcone, sappanchalcone, and 1,8-dihydroxy-4,5-dimethoxy-3-{[(2 s,3r,4 s,5 s,6r)-3,4,5-trihydroxy-6-(hydroxymethyl)oxan-2-yl]oxy}xanthen-9-one and 1,2,4] were detected. The correlation between 5-tetrahydroxy-7-(hydroxymethyl)anthracene-9 and 10-dione was the highest, exceeding 0.99, and the correlation between anthocyanins and peonidin-3-O-(6’’-O-feruloyl)sophoroside-5-O-glucoside was the highest (**Figure 7A**). These results indicated that the anthocyanin content was directly related to the change in the amount of peonidin-3-O-(6’’-O-feruloyl)sophoroside-5-O-glucoside, which regulated the content of peonidin-3-O-(6’’-O-feruloyl). CHI synthesizes sophoroside-5-O-glucoside, which indicates that the CHI gene plays an important role in the synthesis of sweet potato anthocyanin. The correlation between D-fructose 6-phosphate, trehalose 6-phosphate, D-glucose and D-glucose 1,6-bisphosphate was greater than 0.86, and starch had the highest correlation with D-glucose 6-phosphate (**Figure 7B**). These results indicated that the starch content was directly related to the change in D-glucose 6-phosphate content and that the genes regulating D-glucose 6-phosphate were UGP2 and glgA.

### Analysis of differentially expressed TFs

**Figure 8:**
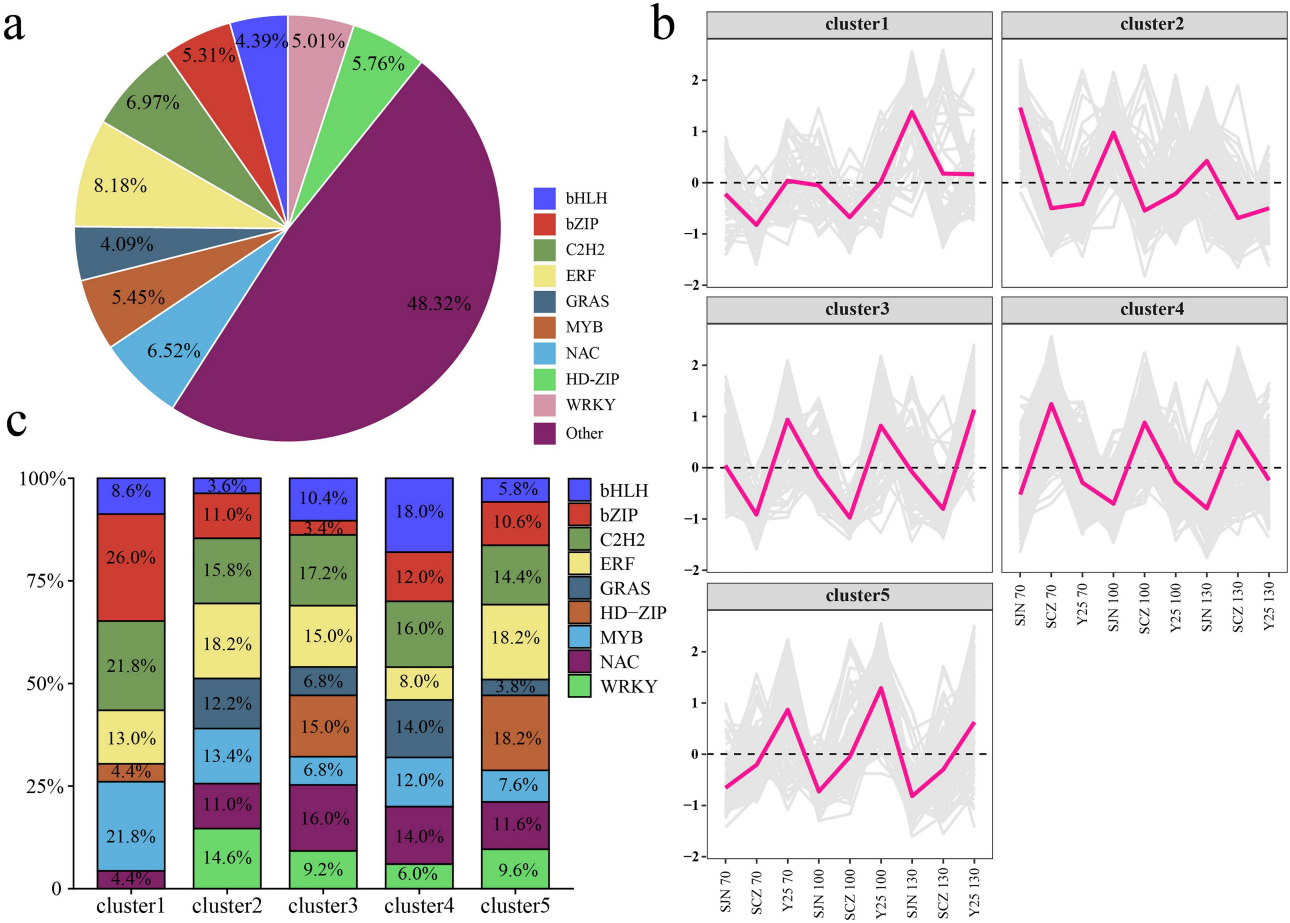
**(A)** Percentage of differentially expressed TFs between cultivars. **(B)** Line chart of the expression patterns of differentially expressed TFs between cultivars. **(C)** Histogram of percentages of different cluster TFs.

TFs are important molecules that control gene expression and are essential for a series of key biological processes. Therefore, we analyzed all the differentially expressed TFs, primarily bHLH (4.39%), bZIP (5.31%), C2H2 (6.97%), ERF (8.18%), GRAS (4.09%), MYB (5.45%), NAC (6.52%), HD-ZIP (5.76%) and WRKY (5.01%) (**Figure 8A**). The expression patterns of the TFs can be categorized into five main clusters. Cluster 1 presented the highest expression in SJN and gradually increased as the sweet potato tuber developed, peaking at 130 d. Cluster 2 also presented the highest expression in SJN but declined progressively with increasing tuber development. The expression of Cluster 3 was highest in Y25, remaining relatively constant throughout sweet potato tuber development. Cluster 4 had the highest expression in SCZ, with a stable expression trend during tuber development. Cluster 5 presented the highest expression in Y25 and gradually decreased as the sweet potato tubers developed (**Figure 8B**). Cluster 1 included primarily bZIP, C2H2 and MYB TFs; Cluster 2 included primarily C2H2, ERF and WRKY TFs; Cluster 3 included primarily C2H2, ERF and NAC TFs; and Cluster 4 included primarily bHLH, C2H2, GRAS and NAC TFs. Cluster 5 included primarily C2H2, ERF and HD-ZIP TFs (**Figure 8C**).

### WGCNA

**Figure 9:**
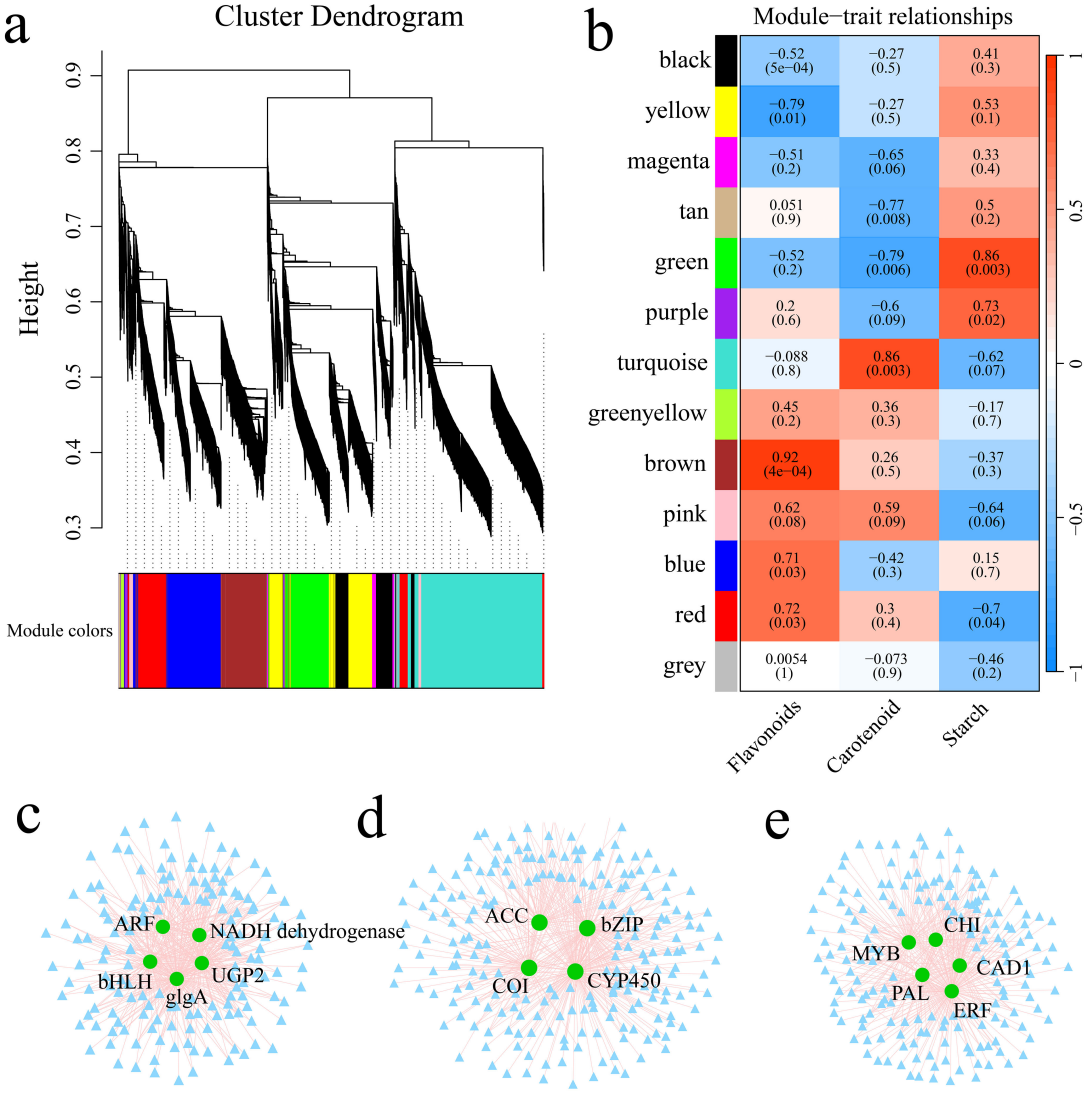
**(A)** Gene hierarchical clustering tree resulting from coexpression network analysis. **(B)** Heatmap illustrating the correlations and significance between modules and the contents of starch, carotenoids, and flavonoids. **(C)** Gene coexpression network within the green module. **(D)** Coexpression network of genes within the turquoise module. **(E)** Gene coexpression network within the brown module.

Utilizing the FPKM values of the genes, the soft threshold calculation identified a value of β=16, which was used to construct the network. The dynamic tree cutting method was applied to merge modules with analogous expression profiles. A total of 13 coexpression modules were generated, with each module represented by a distinct color (**Figure 9A**; **Supplementary Figure S2**). Among the 13 gene modules, the green module was strongly and significantly correlated with sweet potato starch content (r>0.85, p<0.01), the turquoise module was strongly correlated with sweet potato carotenoid content (r>0.85, p<0.01), and the brown module was significantly correlated with sweet potato flavonoid content (r>0.85, p<0.01) (**Figure 9B**). Hub genes typically denote genes with high connectivity within the module. In this study, the kME value (eigengene connectivity) in each module was determined. The gene with the greatest difference was used as the hub gene, and the hub gene and its interacting genes were used to construct a gene interaction network diagram. There were 5 hub genes related to the abundance of starch in sweet potato tubers (**Figure 9C**), 4 hub genes related to the carotenoid content (**Figure 9D**), and 5 hub genes related to the flavonoid content (**Figure 9E**). To further elucidate the relationships between the 14 hub genes and sweet potato anthocyanin, starch and carotenoid, the hub genes were aligned to the *Arabidopsis thaliana* genome using BLAST, and the functions of the core genes were annotated with the help of homologous Arabidopsis genes (**Table 1**). Five genes encoded transcription factors, specifically, ARF, bHLH, bZIP, MYB and ERF. CHI is involved primarily in anthocyanin biosynthesis. NADH dehydrogenase encodes a chloroplast NADH dehydrogenase assembly protein. UGP2 and glgA are involved mainly in starch and glucose biosynthesis. CYP450 and PAL are involved mainly in phenylpropanoid metabolic processes.

**Table 1:** Information on the annotations of candidate genes.

Gene name	Functional annotation
ARF	An important component of auxin signaling
NADH dehydrogenase	Chloroplast NADH dehydrogenase assembly protein
bHLH	Involved in response to light intensity
UGP2	UDP-glucose pyrophosphorylase, functions redundantly with UGP1 for starch biosynthesis during pollen development
glgA	Involved in glucose metabolism
ACC	Regulates rate-limiting enzymes in fatty acid synthesis
bZIP	Regulates the development of flowers and fruits
COI	Jasmonic acid signaling receptor protein
CYP450	Involved in phenylpropanoid metabolic process
MYB	Involved in indole glucosinolate biosynthesis
CHI	Involved in anthocyanin biosynthesis
CAD1	A key enzyme in the lignin synthesis pathway
PAL	Key enzymes in the phenylpropanoid metabolic pathway
ERF	Regulates pigment changes, softening of fruits, and the ripening process of fruits

### qRT−PCR

**Figure 10:**
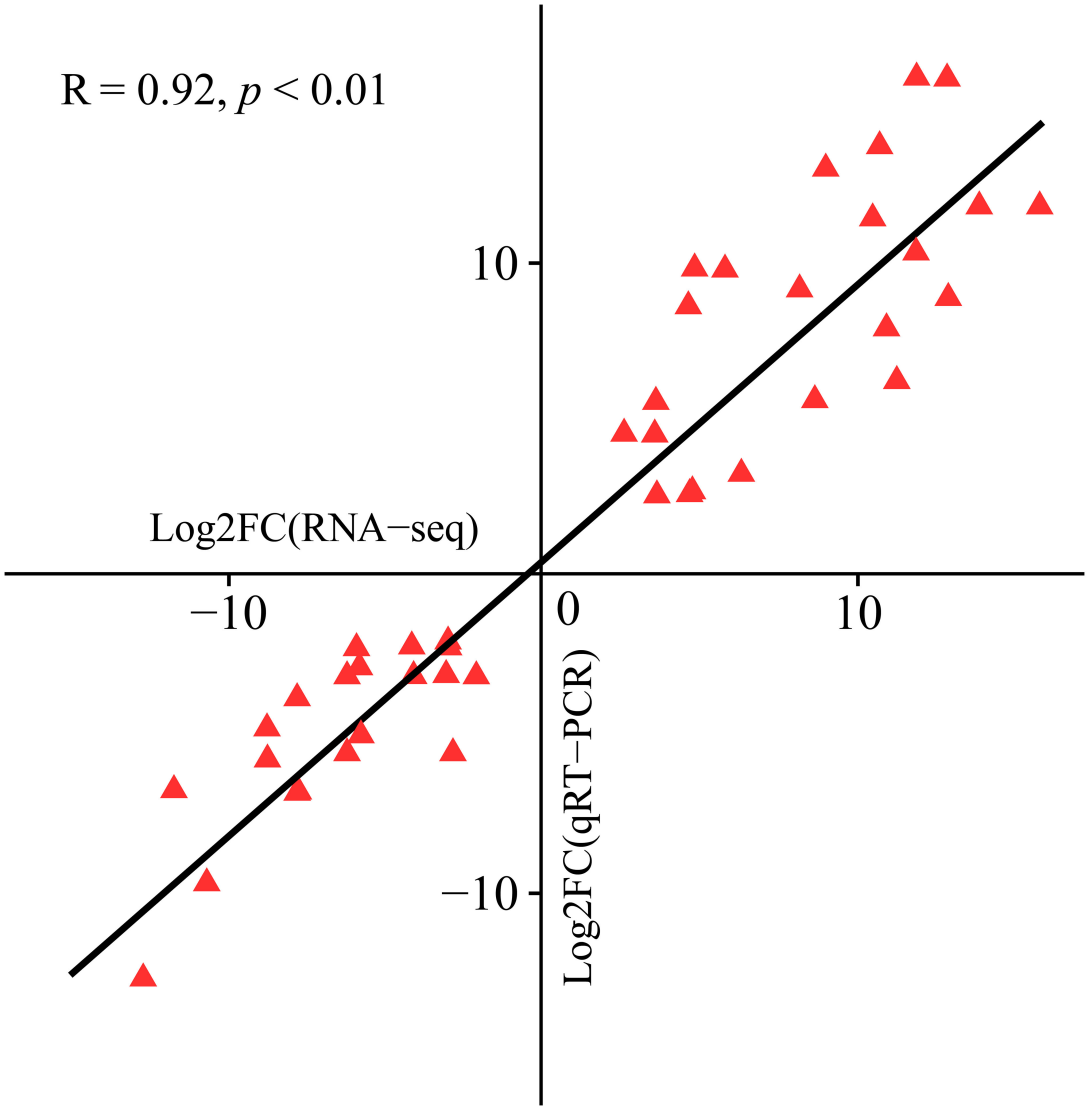
Scatter plot of the correlation between the RNA-seq and qRT−PCR data.

To verify the accuracy of the RNA-seq data, we performed qRT−PCR analysis on 15 hub genes and evaluated their correlation with the RNA-seq data. The findings demonstrated a significant correlation between the RNA-seq and qRT−PCR data (R = 0.92, P < 0.01), confirming the reliability of the RNA-seq data (**Figure 10**).

## Discussion

Sweet potato is an important food, feed and industrial raw material, and improving its yield and quality is highly important for the sustainable development of agriculture and industry (Yang et al., 2023). Sweet potato has the advantages of high nutritional value and wide adaptability of plants, and the study of its biological mechanisms is helpful for its breeding. The tuber of sweet potato plants contain high levels of sugars, phenols and other substances, particularly starch, carotenoids and anthocyanins (Yang et al., 2023; Li Y. et al., 2022; Mohanraj and Sivasankar, 2014; Xu et al., 2018). The main tuber colors are white, orange and purple, so three different representative varieties were selected to determine the anthocyanin, starch and carotenoid contents of their tubers after 70, 100 and 130 d. The white sweet potato had the highest starch content, and the starch content gradually decreased with development of the tuber (**Figure 1**). Starch has always been the most direct demand of the sweet potato starch processing and related industries, so determining the starch content of tubers is a critical breeding goal (Lyu et al., 2021). The orange sweet potato had the highest carotenoid content, and the content of carotenoids gradually increased with the development of tubers, reaching a maximum value at 130 d (**Figure 1**). Sweet potato stems and leaves, particularly stem tips and leaves, are rich in carotenoids, but the content of carotenoids in sweet potato tubers is low and varies greatly (Drapal and Fraser, 2019). We found that the carotenoid content of sweet potato was negatively correlated with the starch content, and increasing the starch content in varieties with high carotenoid content is one of the difficulties in breeding; even if the carotenoid content of the bred varieties is very high, the starch content is too low, and it is difficult to promote and utilize such varieties in production. The purple sweet potato had the highest anthocyanin content, with a slight decrease in the anthocyanin content with the development of the tuber (**Figure 1**). Studies have shown that the biosynthesis of anthocyanins varies greatly in different species, different tissues, and at different developmental stages. Anthocyanin synthesis in plant shoot organs (leaves, flowers, fruits, etc.) requires light, but purple-fleshed sweet potato tubers are underground, completely protected from light, and can still accumulate a large amount of anthocyanins, which indicates that the mechanism of anthocyanin accumulation in the tuber of purple-fleshed sweet potato tubers may differ from that in the aboveground organs of the plant (Li and Ahammed, 2023; Li et al., 2019; Dong et al., 2022).

To further reveal the key genes and metabolites related to anthocyanin, starch and carotenoid contents during the development of sweet potato tubers, RNA-seq and metabolome sequencing were performed on the tubers of SJN, SCZ and Y25 at 70, 100 and 130 d. PCA of the transcriptome metabolomes revealed that the differences between the cultivars were greater than those at different developmental stages within the cultivars, indicating that the differences in gene expression between the cultivars explained the different development of the three cultivars (**Figures 2**, **3**). Starch and sucrose metabolism and flavonoid biosynthesis were coenriched between the DRMs and DEGs, which may be the core pathways related to the anthocyanin, starch and carotenoid contents in sweet potato. Anthocyanins are among the main secondary metabolites involved in plant flavonoid biosynthesis, and the synthesis of plant anthocyanins is regulated mainly by the expression of genes related to anthocyanin synthesis (Tanaka et al., 2008; Sunil and Shetty, 2022; Li Y. et al., 2022). The upstream structural genes involved in anthocyanin synthesis (PAL, C4H, 4CL, CHS, CHI and F3’H) are regulated mainly by MYB regulatory genes, whereas the downstream structural genes (DFR and ANS) are regulated by the WD40/bHLH/MYB complex (Tanaka et al., 2008; Sunil and Shetty, 2022; Li C. et al., 2022). Based on the RNA-seq data, we found that the flavonoid biosynthesis DEGs in sweet potato tuber presented relatively high expression in SCZ, with the highest expression occurring at 130 d, and that SCZ presented a relatively high anthocyanin content. The upstream structural genes (C4H and CHI) of the anthocyanin synthesis pathway were expressed in the tubers of purple-fleshed sweet potato, and the lowest expression levels were detected in white-fleshed and orange-fleshed sweet potato; therefore, their effects on anthocyanin synthesis and metabolism were greater than those of downstream structural genes (DFR and ANS). The network of DEGs and DRMs revealed that *IbCHI* could regulate 11 flavonoid compounds, and *IbC4H* controlled 4 flavonoid compounds, including 2 positive and 2 negative regulatory compounds. PAL is the first enzyme in the flavonoid synthesis pathway and regulates the synthesis of flavonoids such as anthocyanins. We found that the two *IbPAL* genes presented similar expression patterns to those of the upstream structural genes (C4H and CHI) of the anthocyanin synthesis pathway and were expressed mainly in purple sweet potato, which has the highest anthocyanin content. Taken together, these results showed that the synthesis of anthocyanins in sweet potato was regulated mainly by *IbC4H, IbCHI* and *IbPAL* (**Figures 1**, **6**).

As an important food and industrial raw material, the starch of sweet potato tubers has great economic value and importance; thus, the biosynthesis and decomposition mechanisms of sweet potato starch have attracted increasing attention from researchers (Yang et al., 2018; Haque et al., 2023). The mechanism of action of key enzymes responsible for the synthesis and break down of plant starch in metabolic pathways, such as ADP-glucose pyrophosphorylase (AGPase), granule-binding starch synthase (GBSS), glgA, GYS and UPG2, and their effects on the growth period of cash crops have also become popular research topics (Stitt and Zeeman, 2012). At present, with the gradual deepening of research on the starch metabolism pathway, there is a new understanding of the key enzyme-encoding gene sequence, protein structure and function, and related expression regulatory factors in the pathway, particularly in rice, wheat and *Zea mays*, but there are few relevant reports on this topic in sweet potato (He et al., 2021; Schoen et al., 2021; Schultz and Juvik, 2004). In this study, expression analysis revealed that the DEGs related to starch biosynthesis, such as *IbglgA*, *IbGYS* and *IbUPG2*, were more highly expressed in the high starch content variety (SJN), and the highest expression levels were detected at 70 d (**Figure 6B**). In the starch biosynthesis pathway, *IbUGP2* can positively regulate 8 metabolites, and *IbglgA* can also control 8 flavonoids, including 3 positive and 5 negative regulatory compounds (**Figure 6D**). Therefore, *IbglgA, IbGYS* and *IbUPG2* may be important genes for sweet potato starch synthesis and could be key genes for future research.

Sweet potato tubers produce different flesh colors because of the accumulation of different natural pigments, and sweet potatoes are yellow-fleshed, orange-fleshed and orange−red-fleshed because they are rich in carotenoids; these varieties have high nutritional quality and commodity value (Xu et al., 2018). The carotenoids in sweet potato tubers exist mainly in the form of β carotene, lutein, zeaxanthin, vioxanthin and carotenoid intermediate metabolites (Islam et al., 2016). Carotenoids are generally synthesized from the 5-carbon (C5) compound isopentenyl diphosphate ester (IPP), which has two sources: the mevalonate (MVA) pathway in the cytosol and the 2-methyl-D-erythritol-4-phosphate (MEP) pathway in plastids (Ito et al., 2009). Through WGCNA, we found that the turquoise module was highly significantly correlated with the content of sweet potato carotenoids (r>0.85, p<0.01), and there were more genes in the MEP pathway than in the MVA pathway. These findings indicate that sweet potato mainly synthesizes carotenoids through the MEP pathway, and we identified a key gene (*IbGGPS*) of the MEP pathway.

## Conclusions

In this study, the RNA-seq and metabolome of sweet potato tuber at three stages of development were analyzed. DEGs and DRMs were significantly enriched in the starch and sucrose metabolism and flavonoid biosynthesis pathways. DEGs of the flavonoid pathway presented greater expression levels with the development of tubers, with the highest expression occurring at 130 d. DEGs of the starch pathway presented lower expression levels with the development of tubers, with the highest expression level occurring at 70 d. Three specific modules highly related to starch, carotenoids and anthocyanins in sweet potato tubers were identified via WGCNA, and 14 candidate genes were identified. This study elucidated the network mechanism involved in the synthesis and regulation of anthocyanins, starch and carotenoids in sweet potato and lays a theoretical foundation for further analysis of the molecular mechanisms of sweet potato. Although important genes involved in the biosynthesis of sweet potato tuber anthocyanins, starch and carotenoids have been identified, further research is needed on how these genes function and the regulatory relationships among them.

## Data Availability

The data presented in the study are deposited in the NCBI repository, accession number PRJNA1109736.

## Supplementary material

Supplementary Figure 1 Correlation analysis of RNA-seq samples.

Supplementary Figure 2 The scale-free topology fit index as a function of the soft-thresholding power; the red line indicates an R2 value of 0.8.

Supplementary Table 1 All primers used in this study.

Supplementary Table 2 Summary statistics for RNA-seq analysis of 70 d, 100 d and 130 d tuber samples from sweet potato.
