# Supplementary figures and images for: Comparative transcriptome and metabolome analysis of sweet potato (Ipomoea batatas (L.) Lam.) tuber development
Source: Front Plant Sci. 2025 Jan 7;15:1511602. doi: 10.3389/fpls.2024.1511602 (PMC11747047; doi:10.3389/fpls.2024.1511602)

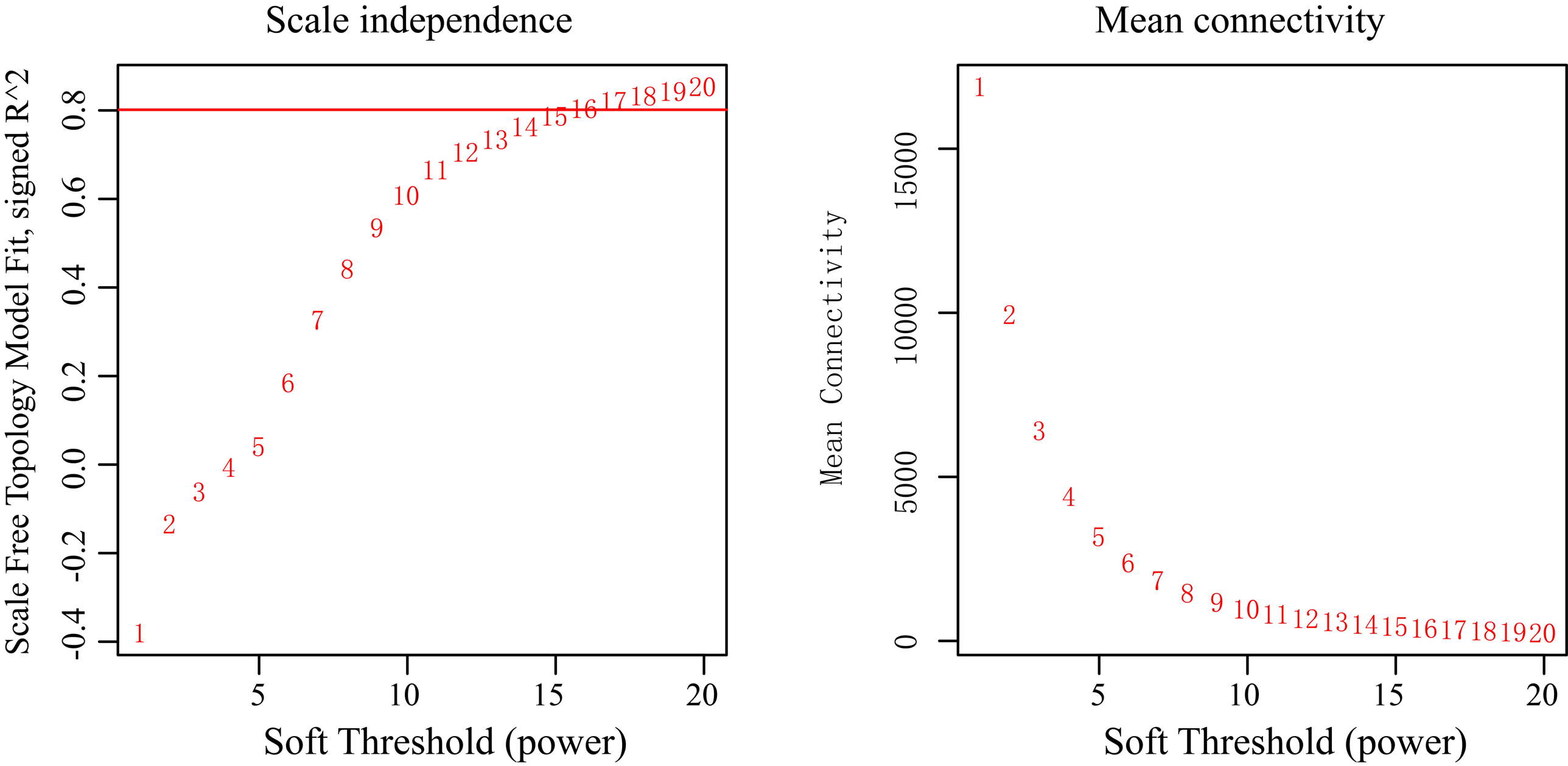

Supplement: Supplementary Figure 2 — The scale-free topology fit index as a function of the soft-thresholding power; the red line indicates an R2 value of 0.8. [file Image2.jpeg]
